# *KRAS*, *BRAF* and *PIK3CA* Status in Squamous Cell Anal Carcinoma (SCAC)

**DOI:** 10.1371/journal.pone.0092071

**Published:** 2014-03-18

**Authors:** Andrea Casadei Gardini, Laura Capelli, Paola Ulivi, Massimo Giannini, Eva Freier, Stefano Tamberi, Emanuela Scarpi, Alassandro Passardi, Wainer Zoli, Angela Ragazzini, Dino Amadori, Giovanni Luca Frassineti

**Affiliations:** 1 Istituto Scientifico Romagnolo per lo Studio e la Cura dei Tumori (IRST) IRCCS, Meldola, Italy; 2 Radiotherapy Unit, Macerata Hospital, Macerata, Italy; 3 Oncology Unit, Degli Infermi Hospital, Faenza, Italy; University of Algarve, Portugal

## Abstract

Anti-EGFR therapy appears to be a potential treatment option for squamous cell anal carcinoma (SCAC). *KRAS* mutation is a rare event in SCAC, indicating the absence of the principal mechanism of resistance to this type of therapy. However, no information is available from the literature regarding the status of *BRAF* or *PIK3CA* in this cancer type. We analysed *KRAS*, *BRAF* and *PIK3CA* status in SCAC patients in relation to the clinical-pathological characteristics of patients and to the presence of the human papilloma virus (HPV). One hundred and three patients were treated with the Nigro scheme for anal cancer from March 2001 to August 2012. Fifty patients were considered for the study as there was insufficient paraffin-embedded tumour tissue to perform molecular analysis the remaining 53. DNA was extracted from paraffin-embedded sections. *KRAS*, *BRAF* and *PIK3CA* gene status and HPV genotype were evaluated by pyrosequencing. *KRAS* and *BRAF* genes were wild-type in all cases. Conversely, *PIK3CA* gene was found to be mutated in 11 (22%) cases. In particular, 8 mutations occurred in exon 9 and 3 in exon 20 of the *PIK3CA* gene. These findings suggest that SCAC could potentially respond to an anti-EGFR drug. *PIK3CA* mutation may be involved in the process of carcinogenesis in some cases of SCAC.

## Introduction

Although anal carcinoma is not a common tumour, its incidence has increased progressively in parallel with transmitted viral infections. Infection from human papilloma virus (HPV) is the main etiologic factor for anal cancer and a high percentage of patients are HPV-positive [1]. Up until 20 or 30 years ago, surgery was the standard treatment for this tumour, consisting in abdominoperineal resection or Miles Operation. The overall 5-year survival is around 50–70%.

In recent years, the therapeutic approach to anal cancer has changed dramatically from demolitive surgery to conservative treatment with radiochemotherapy. Today surgery is mainly used for diagnostic purposes and/or as salvage treatment in locoregional failure after radiochemotherapy.

The first description of the use of radiochemotherapy goes back to the 1970s when the Nigro regimen showed a high rate of complete responses in patients undergoing surgery after preoperative treatment with low doses of radiation (30 Gy) administered in combination with 5-fluorouracil and mitomycin C. Treatment has changed very little since then. Recently, a clinical response to anti-EGFR drugs was observed in single patients [2], [3] and in small case series of patients [4], suggesting their potential effectiveness in this type of cancer.

EGFR expression in anal carcinoma is observed in approximately 80–90% of cases [5]–[7]. Moreover, some studies have demonstrated that *KRAS* mutations, the principal mechanism of resistance to anti-EGFR therapy, are virtually absent in this tumour [5]–[7]. Such information could represent an important prerequisite for the use of anti-EGFR strategies. However, the incidence of other gene alterations, *e.g. BRAF* and *PIK3CA* mutations, involved in the response to anti-EGFR therapies in colorectal cancer [8] has not been studied in anal carcinoma.

In the present study we set out to verify the incidence of *KRAS*, *BRAF* and *PIK3CA* mutations in a series of patients with anal carcinoma and analysed the association between these alterations and the clinical-pathological characteristics of patients.

## Patients and Methods

### Patient Population

We retrospectively analysed 103 patients with SCAC consecutively treated with chemotherapy and radiotherapy at Istituto Scientifico Romagnolo per lo Studio e la Cura dei Tumori in Meldola (Italy) and the Medical Oncology Units of Faenza, Ravenna and Macerata Hospitals (Italy) from March 2001 to August 2012. There was insufficient paraffin-embedded tumour tissue to perform molecular analysis in 53 cases. Our study thus comprised 50 patients with stage I to IV invasive SCAC treated with the Nigro regimen (radiation therapy with concurrent 5-fluorouracil and mitomycin C) for whom paraffin-embedded tumour tissue was available.

According to TNM classification [9], 29 patients had early stage SCAC (stage 1 or 2), 19 patients had stage 3 disease and 2 patients had metastatic cancer. Thirty-four patients had a grade 1 or 2 tumour, and 16 had a grade 3 tumour. Forty-four patients were HIV-negative and 6 were HIV-positive. Thirteen patients had recurrent SCAC.

The study protocol was reviewed and approved by the Medical Scientific Committee of IRST IRCCS, and written informed consent was obtained from patients or from their next of kin for the use of biological samples for research purposes.

### Mutation Analysis

Formalin-fixed paraffin-embedded (FFPE) tumour blocks were reviewed for quality and tumour content. For each case, an area containing at least 50% of tumour cells was selected in hematoxylin and eosin-stained sections, and corresponding 5-μM sections were macrodissected and collected in specific tubes for DNA extraction. Tumour cells were lysed in 50 mM of KCl, 10 mM of Tris-HCl pH 8.0, 2.5 mM of MgCl2 and Tween-20 (0.45%) in the presence of 1.25 mg/ml of proteinase K, overnight at 56°C. Proteinase K was inactivated at 95°C for 10 minutes and the samples were then centrifuged twice at 6000 rpm to eliminate debris. DNA was purified using QIAamp DNA Micro kit (Qiagen, Hilden, Germany) in accordance with the “Genomic DNA clean-up” protocol. DNA quantity and quality were assessed by Nanodrop (Celbio, Milan, Italy).

*KRAS* (exons 2 and 3), *BRAF* (exons 11 and 15) and *PIK3CA* (exons 9 and 20) genes were analysed by pyrosequencing using 3 different kits: anti-EGFR MoAb response (KRAS status), anti-EGFR MoAb response (BRAF status) and anti-EGFR MoAb response (PIK3CA status) (Diatech Pharmacogenetics, Jesi, Italy), according to the manufacturer's instructions. Reactions were run on a PyroMark Q96 ID system (Qiagen).

### HPV detection

HPV DNA was detected in genomic tumour DNA using the HPV sign kit (Diatech Pharmacogenetics), which allows for the amplification and sequencing of a hypervariable region of a highly conserved region of the HPV gene extracted from tumour tissue. Specific genotypes were assigned by pyrosequencing using the Pyromark Q96 ID system (Qiagen) which analyses and aligns the determined sequence with the sequencing library supplied in the kit.

### Statistical Analyses

Progression-free survival (PFS) was calculated from the first day of treatment to the date of the first observation of disease progression or to the date of the last follow-up. Overall survival (OS) was calculated from the first day of treatment to the date of death due to any cause or to the date of the last follow-up. PFS, OS and their 95% confidence intervals (95% CI) were estimated using the Kaplan-Meier life-table method [10] and survival curves were compared by the logrank test [11]. The association between *KRAS*, *BRAF* and *PIK3CA* alteration/mutation and the clinical-pathological characteristics of the patients was analysed by the chi-square test.

Statistical significance was assumed for *P*<0.05 [12]. Statistical analyses were carried out with SAS Statistical software (version 9.3, SAS Institute, Cary, NC).

## Results

Fifty patients (19 males, 31 females) were evaluated. Median age was 62 years (range, 37–87 years). Nineteen (38%) patients had regional nodal involvement, 18 (36.0%) had stage 3–4 disease and 2 (4%) had distant metastases.

All patients had wild type *KRAS* (codons 12, 13, 61 and 146) and *BRAF* (exons 11 and 15), whereas 11 (22%) had a mutation in the *PIK3CA* gene. In particular, 8 had a *PIK3CA* mutation in exon 9 and 3 had a mutation in exon 20 (Table 1). The clinical characteristics of patients are reported in Table 2. All patients were HPV-positive; 46 (92%) had HPV type 16, one had an HPV-16/18 co-infection and the other 3 patients had HPV type 31, 35 or 6.

**Table 1:** Results of PIK3CA mutations.

PIK3CA mutations	No. mutated cases	% Total cases (n = 50)
**All**	11	22
**Exon 9**	8	16
E545K	5	10
E542K	2	4
Q546E	1	2
**Exon 20**	3	6
H1047L	2	4
H1047R	1	2

**Table 2:** Clinical characteristics of the patients with PIK3CA mutation.

	Exon 9	Exon 20
**All patients**	8	3
**Gender**		
Male	2	1
Female	6	2
Age, years		
<60	3	2
≥60	5	1
**Histological grade**		
Grade 1+/Grade 2	3	3
Grade 3	5	0
**Tumour classification**		
1–2	5	2
3–4	3	1
**Lymph node status**		
Negative	5	2
Positive	3	1
**Stage**		
1	2	0
2	3	2
3	4	1
4	0	0
**HIV status**		
Negative	5	3
Positive	3	0
**HPV status**		
HPV-16 positive	8	3
HPV-16 negative	0	0

**Figure 1:**
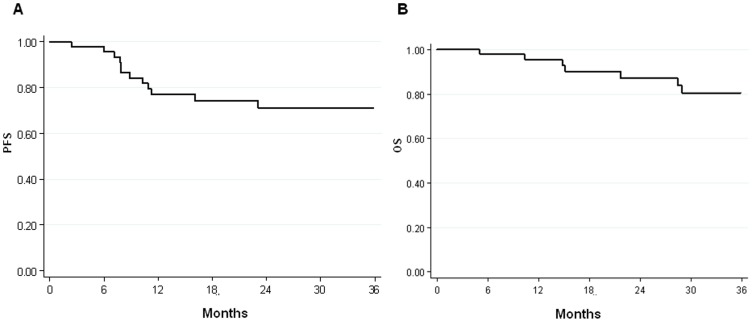
Kaplan-Meier survival curves of (A) progression-free survival (PFS) and (B) overall survival (OS).

At a median follow-up of 38 months (range, 2–138 months), 12 patients had died and 13 patients were in progression. Of these, 7 patients had locoregional failure and 6 had systemic failure. PFS was 77% (95% CI 64–90) at one year and 72% (95% CI 57–85) at 3 years. The overall survival rate was 95% (95% CI 89–100) at 1 year and 81% (95% CI 67–94) at 3 years (Fig. 1). OS and PFS in relation to the clinical-pathological characteristics of patients are summarised in Tables 3 and 4, respectively.

**Table 3:** Progression-free survival in relation to clinical-pathological characteristics of patients.

PFS	No. of patients	No. of events	% 1-year PFS (95% CI)	% 3-year PFS (95% CI)	*P*
**All patients**	50	13	77 (64–90)	72 (57–85)	-
**Type of recurrence**					
Local	7	7	0	0	-
Distant	5	5	40 (0–83)	20 (0–55)	0.042
**Histological grade**					
Grade 1+/Grade 2	34	5	86 (74–99)	82 (68–97)	-
Grade 3	16	8	59 (33–84)	49 (21–76)	0.013
**Tumour classification**					
1–2	27	3	87 (73–100)	87 (73–100)	-
3–4	18	7	68 (45–91)	61 (36–86)	0.026
1–2–3	37	6	84 (71–97)	80 (66–94)	-
4	8	4	58 (22–95)	58 (22–95)	0.031
**Lymph node status**					
Negative	31	6	81 (67–96)	77 (60–93)	-
Positive	19	7	69 (46–92)	61 (37–86)	0.068
**Stage**					
1	8	0	100	100	-
2	21	4	79 (61–97)	79 (61–97)	-
3	19	7	74 (53–96)	57 (31–84)	-
4	2	2	0	0	<0.0001
**HIV** status					
Negative	44	12	74 (60–88)	71 (56–86)	-
Positive	6	1	100	50 (0–100)	0.787
**PIK3CA mutation**					
No	39	10	77 (63–91)	73 (57–88)	-
Yes	11	3	78 (41–100)	65 (32–97)	0.889
**HPV status**					
HPV-16 positive	45	10	82 (69–94)	75 (61–89)	-
HPV-16 negative	5	3	40 (0–83)	40 (0–83)	0.061

**Table 4:** Overall survival in relation to clinical-pathological characteristics of patients.

	No. of patients	No. of events	% 1-year OS (95% CI)	% 3-year OS (95% CI)	*P*
**All patients**	**50**	**12**	**95 (89–100)**	**81 (67–94)**	**-**
**Type of recurrence**					
**Local**	**7**	**7**	**71 (38–100)**	**18 (0–49)**	**-**
** Distant**	**5**	**5**	**100**	**40 (0–83)**	**0.286**
**Histological grade**					
Grade 1 +/Grade 2	34	5	97 (91–100)	88 (74–100)	-
Grade 3	16	7	93 (79–100)	64 (35–93)	0.014
**Tumour classification**					
1–2	27	3	95 (87–100)	89 (75–100)	-
3–4	18	6	94 (83–100)	70 (45–95)	0.054
1–2–3	37	6	97 (90–100)	84 (70–99)	-
4	8	3	87 (65–100)	66 (25–100)	0.231
**Lymphnode status**					
Negative	31	6	100	87 (73–100)	-
Positive	19	6	87 (71–100)	70 (44–95)	0.075
**Stage**					
1	8	0	100	100	-
2	21	4	100	81 (61–100)	-
3	19	7	93 (81–100)	78 (55–100)	-
4	2	1	50 (0–100)	50 (0–100)	0.0002
**HIV status**					
Negative	44	11	95 (88–100)	79 (65–93)	-
Positive	6	1	100	100	0.700
**PIK3CA mutation**					
No	39	9	94 (87–100)	81 (66–97)	-
Yes	11	3	100	75 (45–100)	0.706
**HPV status**					
HPV-16 positive	45	10	91 (88–100)	82 (68–95)	-
HPV-16 negative	5	2	100	75 (33–100)	0.538

**Figure 2:**
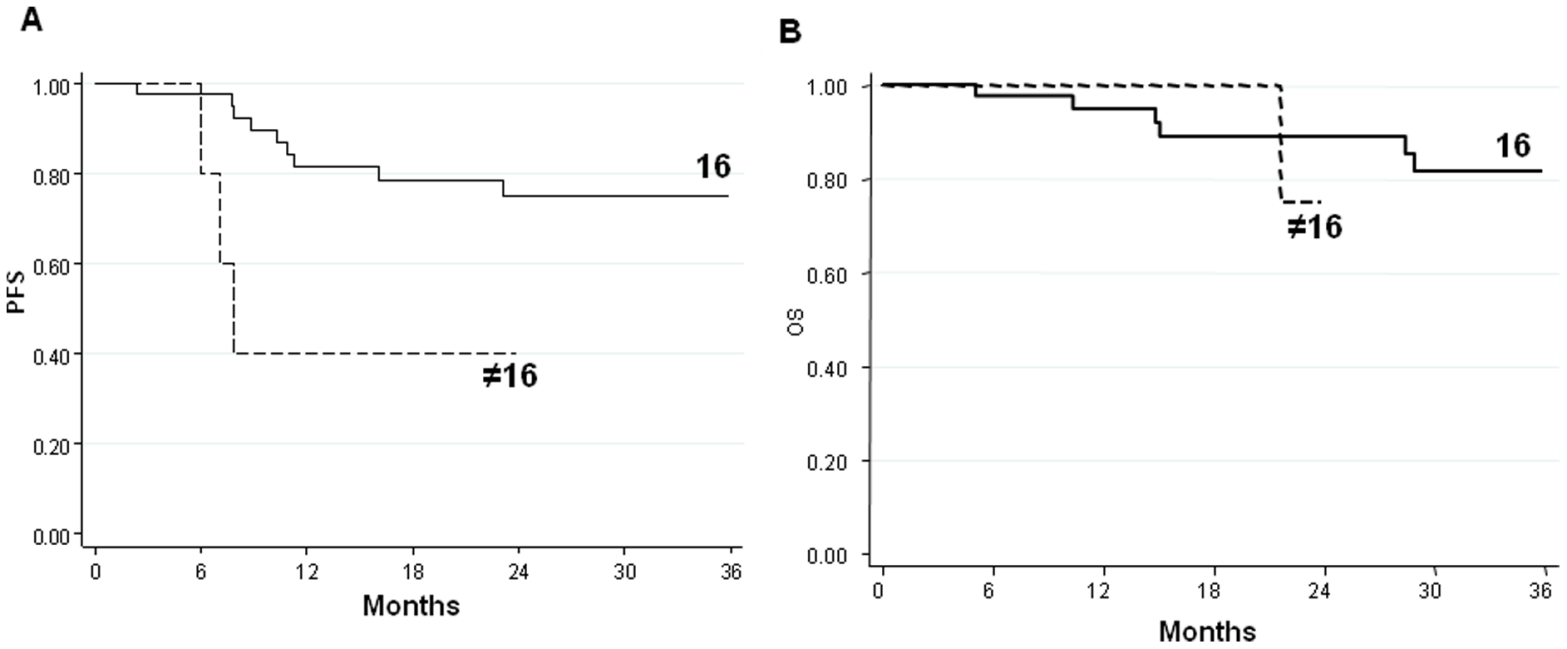
Kaplan-Meier survival curves of (A) progression-free survival (PFS) and (B) overall survival (OS) in relation to HPV-16 status.

No association was found between *PIK3CA* mutations and clinical characteristics of patients. Moreover, no correlation was found between PFS and OS (data not shown). Conversely, HPV-16 was associated with a significantly longer PFS and a higher, albeit not statistically significant, OS compared to the other HPV genotypes (Fig. 2).

## Discussion

Although anal cancer is considered a curable disease, around 20% of patients relapse and a further 30% undergo colostomy for treatment-related toxicities. Treatment has essentially remained the same for 30 years. In our study, PFS and OS were influenced by tumour grade, primary tumour dimension and lymph node status (Fig. 2), in agreement with data presented in the literature [13]. Yhim et al. [14] analysed the correlation between HPV infection and PFS and OS in 48 patients with anal carcinoma. In the HPV-16-positive group, 4-year PFS and OS rates were 63.1% (95% CI, 53.1–73.1) and 84.6% (95% CI, 76.2–93.0), respectively, while 4-year PFS and OS rates in the HPV-16-negative group were 15.6% (95% CI, 5.6–25.7) and 39.8% (95% CI, 26.1–53.4), respectively. HPV-16-positive patients had significantly longer PFS and OS than HPV-16-negative cases (PFS, *P*<0.001; OS, *P* = 0.008). In our study, HPV-16 positive patients had a longer PFS than those with other HPV genotypes, showing a trend towards statistical significance (*P* = 0.061). Such a trend was not observed for OS (*P* = 0.747), even though HPV-16 positive patients showed a longer OS than negative cases.

We did not observe mutations of the *KRAS* gene, in agreement with the findings of other studies [5]–[7]. Moreover, no mutations of the *BRAF* gene were detected. Conversely, 22% of patients were found to have *PIK3CA* exon 9 and exon 20 mutations. No association was found between *PIK3CA* mutations and prognosis. Cetuximab (moAb anti-EGFR) represents a promising treatment choice for anal carcinoma. Several studies on colorectal cancer (CRC) have shown that *KRAS* gene mutations impair response to the drug [15]. It has also been demonstrated that *BRAF* and *PIK3CA* gene mutations in CRC may be associated with a worse response to treatment and poorer prognosis [16]. Our study shows that *KRAS* and *BRAF* are not mutated in anal carcinoma, whereas *PIK3CA*, mutated in about 20% of cases, may represent a mechanism of resistance to anti-EGFR treatment, such as cetuximab. *KRAS* mutation appears to be a rare event in other squamous cell tumours, such as head and neck cancer [17] and cervical cancer [18]. However, the few studies published on *BRAF* mutations in squamous cell carcinomas would seem to indicate a low mutation rate [17]. Recently, some studies reported an incidence of PIK3CA mutations in other squamous cell cancers similar to that observed in our study. In particular, Lui and co-workers found a *PIK3CA* mutation in 30.5% of head and neck cancer patients who were sensitive to anti-mTOR treatment [19]. In a study by McIntyre and co-workers, about 20% of squamous cervical cancer patients showed a *PIK3CA* mutation and these patients had a better prognosis than those with wild-type tumours [20]. These results, together with our findings, suggest that the *PIK3CA* pathway could play an important role in the carcinogenesis of squamous cell carcinoma, especially those related to HPV infection. A more complete understanding of this pathway could facilitate the design of targeted therapy for these types of malignancies.

Two interesting reports were recently published on the use of cetuximab in anal cancer. The first was a phase I study of cetuximab in combination with 5-FU, cisplatin and radiotherapy for locally-advanced anal cancer. Of the 10 patients enrolled, all completed full-dose radiotherapy, 9 were evaluable and 7 (78%) achieved a complete response [21]. The second report focused on the phase II ACCORD 16 study of cetuximab in combination with 5-FU, cisplatin and radiotherapy [22]. Of the 16 patients included in this study, only 8 received the full planned treatment (6 cetuximab administrations and 2 5FU-cisplatin cycles. Accrual was suspended after 10 serious adverse events (6 of which were SUSARs) occurred in 7 of the first 10 patients enrolled. The protocol was amended by decreasing the 5FU and cisplatin dose was decreased and introducing mandatory intensity-modulated radiation therapy. After the accrual of 6 additional patients, a further 6 SAEs occurred in 5 patients. It was thus decided to close the trial due to the high rate of toxicity. Further studies are needed in the neoadjuvant setting to identify acceptable doses of chemotherapy and radiotherapy and thus reduce the risk of severe side-effects. Although it has also been hypothesized that cetuximab could benefit patients with recurrent disease after radiochemotherapy, few studies have been carried out in this area.

In conclusion, despite the relatively small sample size of our study, we believe it is sufficient to demonstrate the presence or absence of the mutations in question. Our results also suggest that the use of the anti-EGFR drug could be used in anal carcinoma, but further studies analysing the effect of this drug in relation to molecular alterations are needed.
